# Umbilical cord coiling: case report and review of literature

**DOI:** 10.1259/bjrcr.20150152

**Published:** 2016-07-22

**Authors:** Rajendra Kumar Diwakar, Meena M Naik, Monika M Jindal

**Affiliations:** ^1^Department of Radiodiagnosis, C.C.M. Medical College & Hospital, Durg, India; ^2^Department of Gynaecology & Obstetrics, C.C.M. Medical College & Hospital, Durg, India

## Abstract

Sonographic evaluation of the umbilical cord coiling can be performed during routine foetal anatomic survey in the second trimester of pregnancy. Umbilical coiling index can be determined by dividing 1 by the intercoil distance in centimetres. Umbilical coiling index has been reported to be around 0.21 + 0.07 (standard deviation) coils per centimetre. Abnormal coiling in its two forms, hypercoiling and hypocoiling, have been reported to be more frequent in gestational diabetes and pre-eclampsia.

## Clinical presentation

### Case 1

Patient aged 32 years, gravida 3 para 3, abortion nil, with a history of amenorrhoea of 7 months duration reported to the department of radiodiagnosis for routine antenatal ultrasound.

Obstetric evaluation of the patient revealed no specific complaints. Her general condition was good. Blood pressure was 120/80 mmHg; pulse 76 min^–1^, regular; temperature was normal; body weight 49 kg. Menstrual history, 3–4/28–30-day cycle, regular.

Abdominal examination revealed 26 weeks size fundal height of the uterus. Blood examination revealed haemoglobin 11.8 g dl^–1^; total leukocyte count 9900 mm^–3^; differential leukocyte count, neutrophils 77%, lymphocytes 18%, monocytes 2%, eosinophils 3%, basophils 0%. Total red blood cells 5.31 million mm^–3^, packed cell volume 36.4%, platelet count 2.25 million mm^–3^; blood urea 14 mg%, serum creatinine 0.64 mg dl^–1^. Human immunodeficiency virus/venereal disease research laboratory/hepatitis B surface antigen were non-reactive; blood group “A” Rh positive; bleeding time and clotting time were normal. Urinalysis was normal.

**Figure 1.**
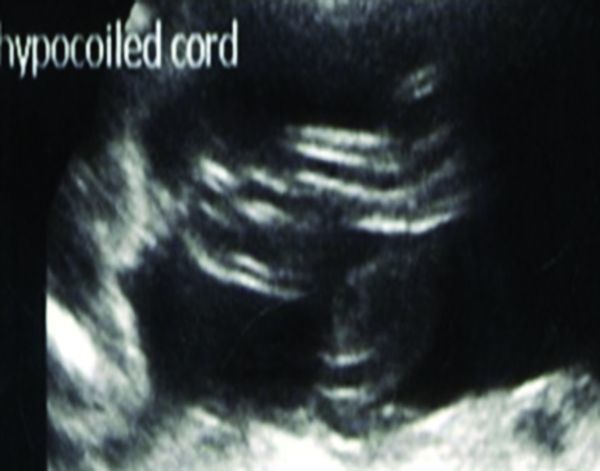
Normal hypocoiled cord at 21 weeks gestation.

**Figure 2.**
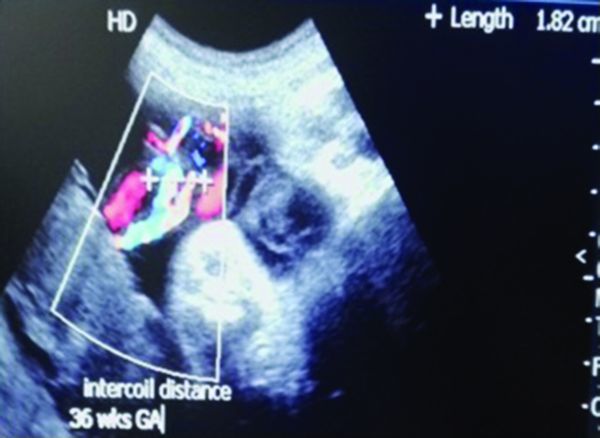
Normal intercoil distance (umbilical coiling index 0.55) at 36 weeks GA. GA, gestational age.

Ultrasound examination revealed a single live intrauterine foetus in the cephalic position of 24 weeks gestational age (GA), while GA by last menstrual period was 31.2 weeks. Foetal movements and cardiac pulsations were present. Amniotic fluid was normal. Placental position was fundal, posterior with maturation grade 0. Estimated foetal body weight was 649 g (50th percentile). The umbilical cord was a three-vessel cord with absence of coiling (Figure 1). Colour imaging revealed an uncoiled umbilical cord (Figure 2).

### Case 2

**Figure 3.**
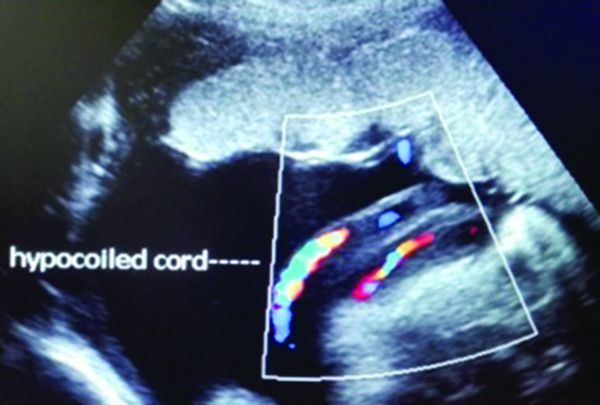
Hypocoiled umbilical cord at 32 weeks pregnancy.

Patient aged 24 years, gravida 4, normotensive, no history of diabetes mellitus, ultrasound examination at 32 weeks GA revealed hypocoiled cord, estimated foetal weight (EFW) 2000 g (Figure 3).

### Case 3

**Figure 4.**
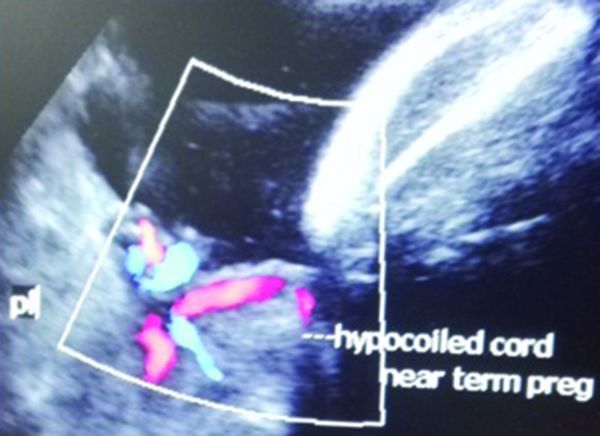
Hypocoiled umbilical cord in the lower segment of the uterus at 36 weeks pregnancy. pl, placenta.

Patient aged 28 years, primigravida, normal blood pressure, no gestational diabetes, revealed hypocoiled cord near term pregnancy (39 weeks GA by ultrasound), EFW 2545 g (Figure 4).

### Case 4

**Figure 5.**
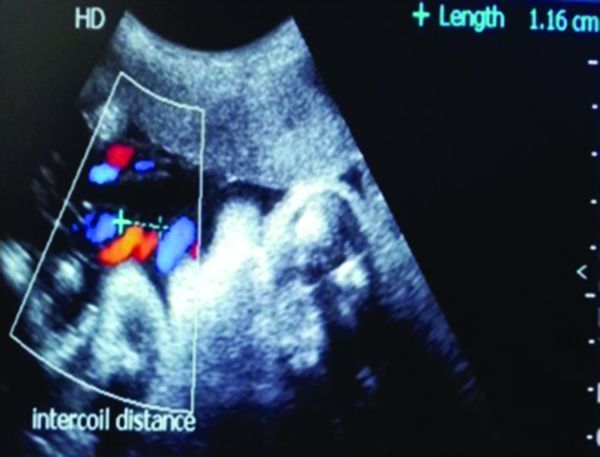
Measurement of intercoil distance (umbilical cord index 0.86) in a hypercoiled umbilical cord.

Patient aged 22 years, gravida 2 with normal blood pressure, no diabetes mellitus, nothing significant in past obstetric history, ultrasound examination at 36 weeks GA revealed hypercoiled cord; EFW 2250 g (Figure 5).

## Discussion

**Figure 6.**
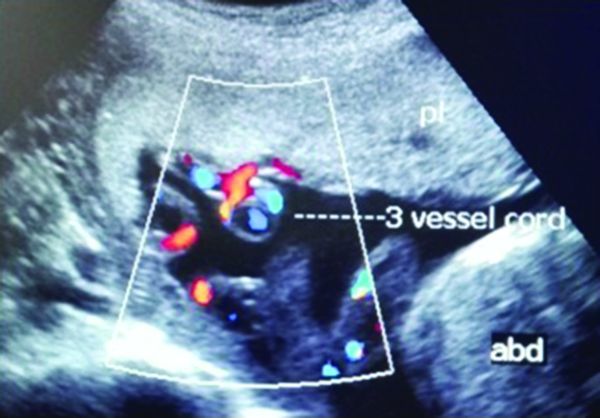
A three-vessel (two arteries and one vein) normal umbilical cord in cross-section. abd, foetal abdomen; pl, placenta.

**Figure 7.**
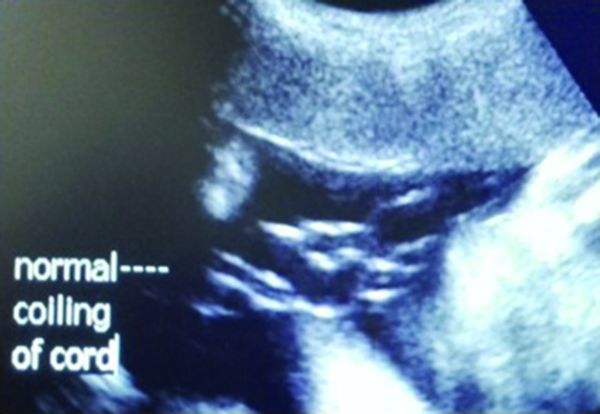
Normal coiling of the umbilical cord in the third trimester of pregnancy.

The normal umbilical cord extends from the placenta to the foetal umbilicus, twisting (coiling) as it traverses the distance between these two points. The umbilical cord normally contains two umbilical arteries and an umbilical vein (Figure 6) that are coiled (Figure 7). Hypocoiled or hypercoiled cords are associated with increased foetal morbidity, including small for GA foetuses, risk for pre-term delivery, low birth weight neonates and adverse perinatal outcomes.

The umbilical cord and its vital blood vessels are the most vulnerable part of the foetal anatomy. The pattern of coiling develops during the second and third trimesters, presumably due to snarls in the cord, and this coiling changes as the pregnancy advances.^1^

The mechanism by which physiologic coiling occurs still remains undetermined, with speculation that it may be related to early foetal activity and haemodynamic factors, or other anatomical issues such as the presence of Roach muscle.^2^

Normally the umbilical cord coils to the left. Regardless of its origin, umbilical coiling appears to confer turgor to the umbilical unit, producing a cord that is strong but flexible^3^ The role of umbilical cord coiling is not clear; nonetheless, it is thought to play a role in protecting the umbilical cord from external forces such as tension, pressure, stretching or entanglement.^4^

It is believed that abnormal cord coiling is a chronic state, established during early gestation, that may have chronic (growth retardation) and acute (foetal intolerance to labour and foetal demise) effects on foetal well-being. Yet, the cause of abnormal cord coiling remains unknown in a majority of cases.^4^

Abnormal cord coiling in its two forms, hypercoiling and non-coiling, has been reported to be more frequent in females with gestational diabetes and pre-eclampsia. A higher frequency of thrombosis of chorionic plate vessels, umbilical venous thrombosis and cord stenosis has been reported.^4^

Wharton’s jelly is a gelatinous substance within the umbilical cord, which is largely made up of mucopolysaccharides. It also contains some fibroblasts and macrophages. Being a mucous tissue, it protects and insulates the umbilical blood vessels. In Wharton’s jelly, the most abundant glycosaminoglycan is hyaluronic acid, which forms a hydrated gel around the fibroblasts and collagen fibrils and maintains the tissue architecture of the umbilical cord by protecting it from pressure.^5^

The second trimester extends from 13 to 27 weeks 6 days of gestation. 22–28 weeks is considered the most suitable time for measurement of umbilical coiling.^6^ The measurement of the umbilical cord should be taken near the placenta or in the middle segment. The distance between the coils should be measured from the inner edge of an arterial or venous wall to the outer edge of the next coil along one side of the umbilical cord. In the third trimester of pregnancy, the volume of amniotic fluid is reduced, resulting in errors of measurement of coiling of the cord.

Morphology of the umbilical cord shows marked changes depending on GA. This is particularly true for the first trimester of gestation, in which the umbilical cord vessels change rapidly from a predominantly parallel to the typically twisted appearance. It is interesting that maturation of the umbilical cord morphology occurs with other important events such as formation of the intervillous space and appearance of diastolic flow velocities in umbilical arteries Doppler waveforms. A normal umbilical cord contains two arteries and one vein.

This incidence sharply decreases with advancing gestation and after 14 weeks of gestation, only 9.6 ± 5.8% of umbilical cords are classified as uncoiled.^7^

The frequency of non-coiled cords and poorly coiled cords is approximately 4% to 5%.^8^ It has been suggested that non-coiled cords are structurally less able to resist external forces. Non-coiled cords are associated with intrauterine death, pre-term delivery, repetitive intrapartum heart rate decelerations, operative delivery owing to foetal distress, meconium staining, aneuploidy and intrauterine growth restriction (IUGR).

The frequency of hypercoiled cords has been reported to be as high as 21%.^9^ Hypercoiled cords are associated with foetal demise, foetal intrapartum distress, IUGR, chorioamnionitis and nuchal cord loops.

In 300 singleton pregnancies with absence of gross foetal anomalies, Predanic et al^9^ measured the UCI in late second trimester of pregnancy (antenatal UCI) and the true UCI at birth and found a good correlation, with sensitivity values to predict hypocoiling and hypercoiling at birth being 78.9% and 25.4%, respectively.

The umbilical coiling was first quantified by Edmonds,^1^ who divided the total number of coils by the umbilical cord length in centimetres and called it the “index of twist”. Later, Strong et al^10^ named it the “umbilical coiling index”. Degani et al^11^ suggested the formula (antenatal UCI = 1/distance between two adjacent coils) to calculate the UCI of the umbilical cord floating in the amniotic fluid.

The percentile of umbilical cord coiling is described as: (a) hypercoiled cords (UCI > 90th percentile); (b) hypocoiled cords (UCI < 10th percentile). The mean UCI is 0.24 + 0.09.

It has been reported that both hypocoiled and hypercoiled cords were associated with foetal growth retardation and non-reassuring foetus status in labour; nonetheless, they were not associated with meconium-stained amniotic fluid, interventional delivery, GA at birth, mode of delivery and low Apgar score.^12^

IUGR has many causes, including placental insufficiency, which may be primary (most common cause) or due to maternal disorders such as hypertension, collagen vascular disease, renal disease, poor nutrition, and drug or alcohol abuse. Foetal factors of IUGR include viral infections (*e.g.* cytomegalovirus or toxoplasmosis) and foetal chromosomal anomalies.

Rana et al,^7^ Predanic et al^12^ and Jo et al^6^ reported in their study the association of hypercoiling of the umbilical cord during the late second trimester of pregnancy with the increased risk for pre-term delivery, higher number of low birth weight neonates, and admission to the neonatal intensive care unit.

Several studies^2,8,10,13,14^ have evaluated the antennal and postnatal UCIs and reported poor perinatal outcomes. Risk factors for hypercoiling were extremes of maternal age; and for non-coiling were obesity, gestational diabetes mellitus and pre-eclampsia. Hypercoiled and non-coiled cords were significantly associated with adverse perinatal outcomes and caesarean delivery.^4^

### Limitations of technique

1. Sonographic evaluation of the umbilical cord is an operator-dependent technique.
2. UCI is difficult to calculate in oligohydramnios and in obese patients.
3. Not all hypocoiled or hypercoiled umbilical cords are abnormal.

## Learning points

1. Low UCI is an indicator of adverse perinatal outcome.
2. It is associated with low Apgar score, meconium staining, and pregnancy-induced hypertension.
3. Therefore, it can be helpful in identifying foetuses at risk.

## Consent

Informed consent from the patients has been obtained.
